# Directed Gradients in the Excited-State Energy Landscape of Poly(3-hexylthiophene) Nanofibers

**DOI:** 10.1021/jacs.3c02117

**Published:** 2023-06-14

**Authors:** Sebastian Stäter, Felix A. Wenzel, Hannes Welz, Klaus Kreger, Jürgen Köhler, Hans-Werner Schmidt, Richard Hildner

**Affiliations:** † Zernike Institute for Advanced Materials, University of Groningen, Nijenborgh 4, 9747 AG Groningen, The Netherlands; ‡ Spectroscopy of Soft Matter, University of Bayreuth, Universitätsstrasse 30, 95440 Bayreuth, Germany; § Macromolecular Chemistry I and Bavarian Polymer Institute, University of Bayreuth, Universitätsstrasse 30, 95440 Bayreuth, Germany; ∥ Bavarian Polymer Institute and Bayreuther Institut für Makromolekülforschung (BIMF), University of Bayreuth, 95440 Bayreuth, Germany

## Abstract

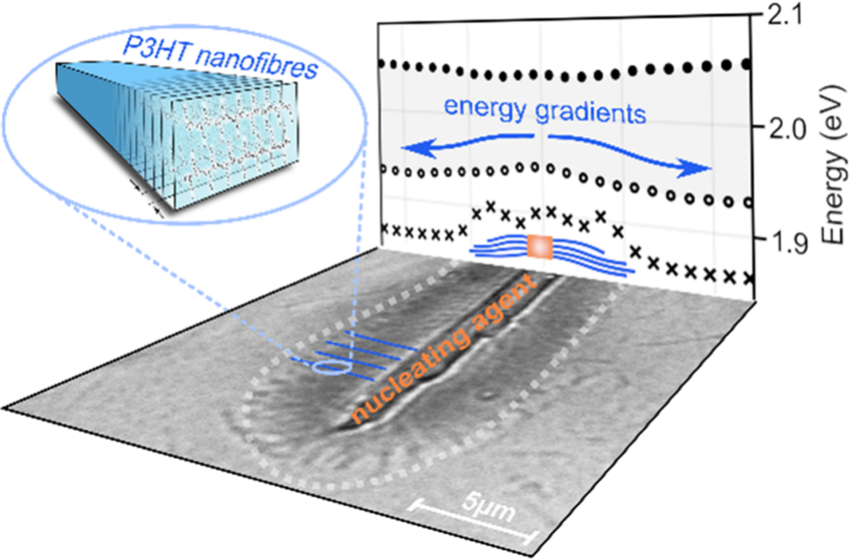

Funneling excitation energy toward lower energy excited states is a key concept in photosynthesis, which is often realized with at most two chemically different types of pigment molecules. However, current synthetic approaches to establish energy funnels, or gradients, typically rely on Förster-type energy-transfer cascades along many chemically different molecules. Here, we demonstrate an elegant concept for a gradient in the excited-state energy landscape along micrometer-long supramolecular nanofibers based on the conjugated polymer poly(3-hexylthiophene), P3HT, as the single component. Precisely aligned P3HT nanofibers within a supramolecular superstructure are prepared by solution processing involving an efficient supramolecular nucleating agent. Employing hyperspectral imaging, we find that the lowest-energy exciton band edge continuously shifts to lower energies along the nanofibers’ growth direction. We attribute this directed excited-state energy gradient to defect fractionation during nanofiber growth. Our concept provides guidelines for the design of supramolecular structures with an intrinsic energy gradient for nanophotonic applications.

## Introduction

The precise flow of excitation energy between nanoscopic functional units is a key step in the initial light-driven steps in photosynthesis. Those functional units comprise light-harvesting complexes that act as antennae and reaction centers that act as transducers. Both units are often complex superstructures with very few chemically different pigment molecules densely packed and precisely arranged within a protein scaffold.^1−4^ Electronic Coulomb interactions between pigments and noncovalent interactions between pigments and the protein scaffold create a funnel, or gradient, in the excited-state energy landscape, i.e., a decreasing energy of the lowest-energy excited states toward the reaction center. This energy gradient provides the driving force to steer excitation energy in a directed and highly efficient way toward the reaction center, where excitation energy is converted into chemical energy.^1,2,5^

Several approaches have been developed to establish (step) gradients in artificial systems. The aim is to transfer energy between molecules or assemblies of molecules in a cascade-type fashion from higher to lower transition energies. Such systems are based on, e.g., thin films comprising different conjugated polymers^6^ or laser dyes,^7^ or a series of up to five chemically different chromophores with decreasing transition energies, which are covalently linked to a strand of DNA.^8^ Supramolecular assemblies have recently attracted great attention as artificial light-harvesting systems as well. For instance, donor–acceptor-type assemblies with various morphologies were designed, such as wire-like structures forming organogels^9^ or sheet-like structures of clay–dye-based hydrogels,^10^ micelles,^11,12^ vesicles,^13,14^ spherical aggregates,^15^ nanofibers and nanotubes.^16−18^ Moreover, distinct BODIPY derivatives that self-assemble into sheet-like morphologies with specific packing and order of the molecules were used to create a “cascaded” excited-state energy landscape between the supramolecular assemblies.^19^

However, concepts that employ only a single chemical species of functional molecules to establish a continuous excited-state energy gradient have not been realized yet to the best of our knowledge. In principle, along one-dimensional supramolecular nanofibers, a continuous shift of the lowest-energy excited state can be achieved by a (increasing or decreasing) trend in intermolecular Coulomb interaction. Using small, rigid, conjugated molecules as building blocks, however, such variation in interaction may be challenging to achieve. The required continuous change in intermolecular distance and/or mutual orientation is impeded by the supramolecular motif(s) and the self-assembly conditions, both of which determine the mutual arrangement of molecules in thermodynamic equilibrium.^20^ In contrast, the crystallization of conjugated polymers from solution results in the incorporation of an increasing number of defects along a nanostructure, thus providing a gradient in defect density. Defects that are relevant in this context are regio-defects of polymer chains, chain ends incorporated within a nanostructure, and intra-chain torsional disorder. Intra-chain torsional disorder refers to a deviation from backbone planarity by a rotation of monomers out of plane. A regio-defect is a local chemical deviation from the polymer structure, typically a mis-attached side chain. Such regio-defects typically increase torsional disorder along polymer chains due to steric hindrance, which influences the polymers’ packing within nanostructures.^21,22^ Similarly, the end of a polymer chain being incorporated into a nanostructure impacts the overall packing;^23^ often, the chain end involves a regio-defect, too. Importantly, such defects do not act as (emissive) trap states for electronic excitations; yet, an increasing defect density can be expected to modulate the excited-state energy landscape along the growth direction of conjugated polymer nanostructures via an increasing degree of disorder in chain packing, thus modulating inter-chain electronic interactions. For instance, Roehling et al. and Oosterbaan et al. showed for polythiophenes such fractionation according to molecular weight and probably regio-defects, implying an increasing number of defects, during growth of supramolecular nanofibers.^24,25^ However, an energy gradient could not be demonstrated.

Here, we exploit defect fractionation during the growth of nanofibers based on a conjugated polymer to realize a gradient in the excited-state energy landscape over micrometer distances. We employ a highly efficient ribbon-like supramolecular nucleating agent (NA)^26^ for the controlled crystallization of the extensively studied and well-understood poly(3-hexylthiophene), P3HT,^27−33^ into nanofibers. The resulting NA/P3HT superstructures, resembling shish-kebab-like structures,^34,35^ with their highly aligned and oriented micrometer-long P3HT nanofibers allow us to use hyperspectral imaging, i.e., spatially resolved absorption and emission spectroscopy. We reveal a continuous red shift of the lowest-energy exciton states exceeding thermal energy at room temperature along the growth direction of the P3HT nanofibers.

## Results and Discussion

**Scheme 1:**
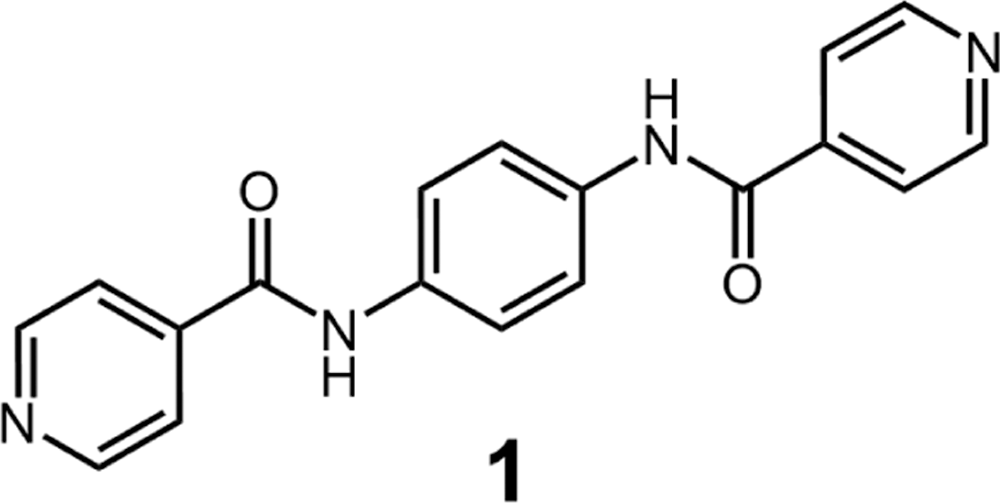
Chemical Structure of *N*,*N*′-1,4-Phenylenebis[4-pyridinecarboxamide], Compound **1** The molecular design combines the ability to form stable supramolecular aggregates via hydrogen bonding of the amide linkages, resulting in epitaxial surfaces with the ability to form attractive pyridine–thiophene interactions to nucleate P3HT.^26^

To demonstrate the concept of defect fractionation on the excited-state energy landscape of supramolecular P3HT nanofibers, knowledge of the starting point and growth direction of nanofibers is essential. Heterogeneous nucleating agents (NAs) are known to provide an epitaxial surface from which the polymer crystallization is initiated, and subsequently, crystal growth proceeds in a defined manner.^36,37^ Achieving very densely packed and oriented nanofibers of P3HT requires NAs with a highly regular surface and a large number of nucleation sites. In our recent work, we have shown that a supramolecular NA based on *N*,*N*′-1,4-phenylenebis[4-pyridinecarboxamide] (compound **1**, see Scheme 1) is an excellent nucleating agent for the oriented crystallization of P3HT.^26^

We employ a two-step protocol to grow P3HT nanofibers as part of a supramolecular superstructure (as detailed in the Supporting Information (SI), Section S1). In the first step, we grow ribbon-like supramolecular NAs based on compound **1**. The self-assembly of compound **1** into supramolecular structures is achieved by controlled heating and cooling of compound **1** in chlorobenzene. The formation of ribbon-like structures with lengths of several tens of μm and widths of 1–5 μm is confirmed by electron microscopy (see SI Figure S2a). In the second step, we add P3HT to a dispersion of the supramolecular NA. The sample is kept for several days at room temperature, allowing P3HT to trans-crystallize from the epitaxial surface of the ribbon-like supramolecular NA into oriented nanofibers. The presence of oriented P3HT nanofibers grown from supramolecular NAs is confirmed via electron microscopy (see SI Figure S2c). Importantly, the P3HT nanofibers are densely packed and highly oriented, with known starting point and growth direction, and lengths of several micrometers. This allows for the straightforward and unambiguous investigation of the nanofibers’ optical and electronic properties as a function of position along their growth direction.

**Figure 1:**
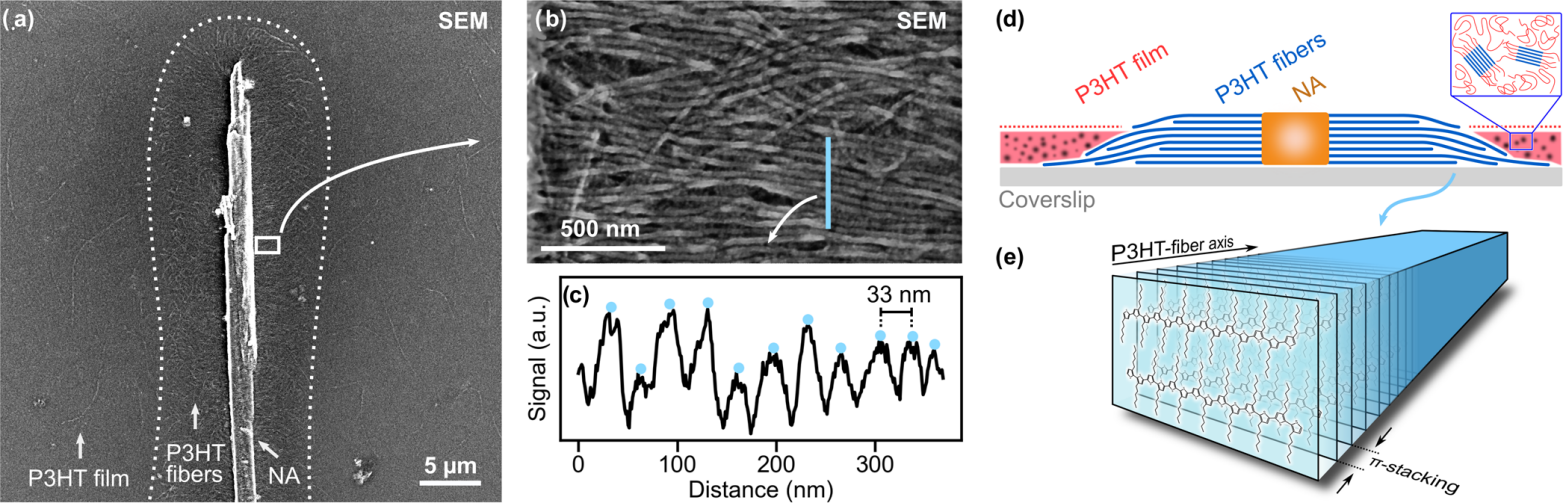
Structural characterization of P3HT nanofibers within a nucleating agent (NA)/P3HT superstructure. (a) Scanning electron microscopy (SEM) image of a NA/P3HT superstructure with a vertically oriented supramolecular NA and P3HT nanofibers that grew perpendicularly from the NA. The dotted white line indicates the area covered by P3HT nanofibers. (b) Magnified view of the P3HT nanofibers within the boxed area in panel (a), highlighting their high degree of orientation. (c) Gray scale profile along the blue line in panel (b); the blue dots indicate the interlamellar distances of ∼33 nm between P3HT nanofibers. (d) Schematic cross section of the NA/P3HT superstructure. The inset illustrates the structure of the semicrystalline P3HT film surrounding the P3HT nanofibers. (e) Schematic view of the packing of P3HT chains into an individual P3HT nanofiber.

**Figure 2:**
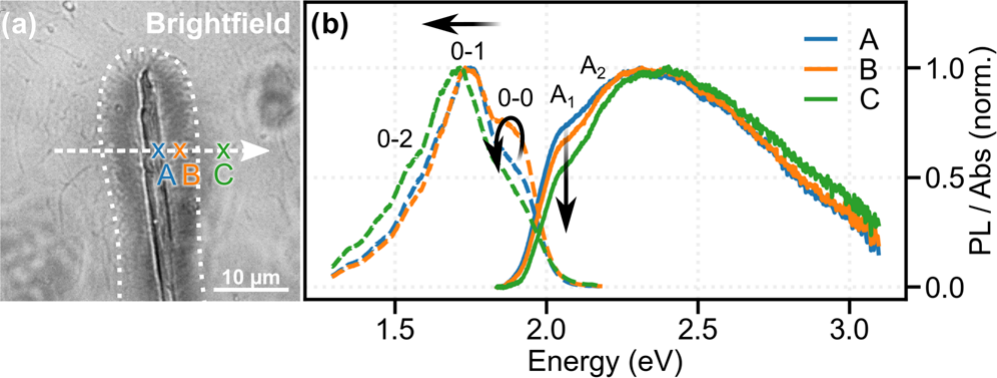
Optical microscopy and spectroscopy of the NA/P3HT superstructure shown in Figure 1. (a) Optical bright-field image. The dotted white line indicates the area covered by P3HT nanofibers as in Figure 1a; the white arrow indicates the direction of scanning for hyperspectral imaging. (b) Examples of spatially resolved PL (dashed lines) and absorption spectra (solid lines) taken at spot A (beginning of P3HT nanofibers), B (middle of P3HT nanofibers), and C (surrounding P3HT film). The arrows indicate spectral changes for increasing distance to the supramolecular NA: a decrease in the lowest-energy absorption A_1_ around 2.1 eV, a red shift of the PL spectra, and the complex behavior of the electronic (0–0) PL peak intensity.

The morphology of NA/P3HT superstructures was characterized by correlative electron and optical microscopy (SI, Section S1). Figure 1a shows a scanning electron microscopy (SEM) image of a representative example of a superstructure. The ribbon-like supramolecular NA is oriented vertically in this figure and features a length exceeding 40 μm and a width of 3 μm. The P3HT nanofibers are oriented horizontally with a dense packing and extend on both sides up to 5 μm away from the central supramolecular NA. The area covered by P3HT nanofibers is indicated with the dotted line in Figure 1a. Figure 1b shows a magnified view of the boxed area in Figure 1a close to the supramolecular NA. Individual P3HT nanofibers are clearly discernible and are arranged with a high degree of parallel alignment with respect to each other (although some nanofibers on the surface are not perfectly aligned). A grayscale profile across the P3HT nanofibers reveals an average distance of 33 nm between signal maxima, as indicated by the blue dots in Figure 1c. This lamellar distance comprises the width of the crystalline P3HT nanofibers, as well as the width of amorphous interlamellar zones between nanofibers. For high-molecular-weight P3HT, as used here, the width of the interlamellar zones is in the range of 10–15 nm. Hence, we estimate that the P3HT nanofibers have a crystalline width of about 20 nm, in agreement with literature data.^27,34,38−41^ A schematic illustration of the NA/P3HT superstructure’s cross section is shown in Figure 1d with the supramolecular NA in orange and the P3HT nanofibers in blue; the π-stacking of P3HT within nanofibers is displayed in Figure 1e (see also SI, Section S2). Residual molecularly dissolved P3HT in solution leads to the formation of a polycrystalline P3HT film (Figure 1d, red) surrounding the NA/P3HT superstructure upon deposition. The optical bright-field microscopy image of the same superstructure in Figure 2a closely matches the SEM image (Figure 1a). Even though individual P3HT nanofibers cannot be resolved optically, the area covered by nanofibers (indicated by the dotted line) is clearly discernible by the lower (but constant) transmission in this region. While the end of P3HT nanofibers may be immersed in or under the P3HT film, the good agreement between the electron and optical microscopy images ensures an accurate assessment of the nanofiber length of about 5 μm.

We reconstruct the excited-state energy landscape along the highly oriented P3HT nanofibers by hyperspectral imaging of the 40 × 40 μm^2^ area shown in Figures 1a and 2a^26,42^ (see the SI, Section S1). Collecting absorption and photoluminescence (PL) spectra with diffraction-limited resolution and moving the sample both horizontally and vertically results in a set of 1680 absorption and 1680 PL spectra fully characterizing our sample. To introduce the analysis procedure and to discuss the qualitative trends, we start with three representative positions: At the beginning (spot A), in the middle of the P3HT nanofibers (spot B), as well as within the surrounding P3HT film, remote from the superstructure (spot C, see Figure 2a). We will later extend this discussion to a horizontal line scan along the P3HT nanofibers as well as to a full representation of the excited-state energy landscape in 2-dimensional 40 × 40 μm^2^ maps. We note that the supramolecular NA does not absorb in the visible range and, thus, the absorption and PL spectra stem exclusively from P3HT.

The absorption spectra from positions A, B, and C (Figure 2b, solid lines) share the same general shape that is characteristic of semicrystalline P3HT.^43^ The structured shoulder in the low-energy part (1.9–2.2 eV), labeled with A_1_ and A_2_, stems from absorption into exciton states delocalized along π-stacks of P3HT chains forming H-aggregates. The broad, featureless shape in the high-energy region (2.3–3.1 eV) results from the absorption of amorphous P3HT chains. These spectra thus confirm the coexistence of crystalline and amorphous P3HT both in the nanofiber region and the surrounding P3HT film. The main difference in the absorption spectra is the relative intensity of the lowest-energy shoulder A_1_ at 2.1 eV, which indicates variations in the electronic Coulomb interaction *V* between π-stacked chains as a function of position.^43^ Specifically, when going from the beginning (spot A) toward the middle of the P3HT nanofibers (spot B) and into the surrounding P3HT film (spot C), the decreasing relative intensity of this shoulder suggests an increasing inter-chain electronic interaction.

To quantify the change in electronic interaction between π-stacked P3HT chains as a function of position, we exploit the Frenkel Polaron model.^44^ This model describes the optical properties of molecular H-aggregates in the presence of intra-molecular vibrations and has been widely applied to aggregates of conjugated polymers, such as P3HT.^43^ By fitting the low-energy part of the absorption spectra (SI, Section S3), we extract the free exciton bandwidth *W*, which relates to the electronic interaction *V* between P3HT chains via *W* = 4*V*. When going from the beginning of the P3HT nanofibers (spot A) toward their middle (spot B) and into the P3HT film (spot C), we find from the fits to the spectra in Figure 2b that the free exciton bandwidth increases from 193 to 248 meV (Table 1). While this range of values is in agreement with literature data,^29,31,43^ the substantial change of this parameter within the same sample (in fact, within a few micrometers of our sample) is remarkable. Along the same direction (from A to C), the spectral position of the lowest-energy absorption peak A_1_ of the crystalline phase *E*_A1_^Abs^ blue-shifts by 10 meV, starting from 2.043 eV at spot A (Table 1).

**Table 1:** Free Exciton Bandwidth *W* and Spectral Position of the Lowest-Energy Absorption Peak *E*_A1_^Abs^ and of the Electronic 0–0 PL Peak *E*_00_^PL^ for the Spectra of P3HT Aggregates in Figure 2b^a^

	spot A (start of nanofibers)	spot B (middle of nanofibers)	spot C (surrounding film)
*W* (meV)	193	211	248
*E*_A1_^Abs^ (eV)	2.043	2.044	2.054
*E*_00_^PL^ (eV)	1.903	1.904	1.862

a The positions of spots A, B, and C are indicated in Figure 2a.

A straightforward explanation for the increasing trend of the exciton bandwidth, and thus of the increasing inter-chain electronic interaction along P3HT nanofibers, would be a decrease in the π–π-stacking distance between P3HT chains. However, selected-area electron diffraction (SAED; see the SI, Section S2) demonstrates that this distance does not change substantially along our P3HT nanofibers. In π-stacked, H-type aggregates of conjugated oligomers and polymers a further effect plays a strong role for the exciton bandwidth. For a given π-stacking distance, the inter-chain electronic interaction increases if the delocalization of electronic excitations within a chain decreases.^24,31,45^ Such decreasing intra-chain delocalization can be caused by the presence of more and more torsionally disordered chain segments. This disorder, in turn, originates from an incorporation of an increasing number of chain-end and regio-defects during nanofiber growth, i.e., from the start (spot A) toward the end of our P3HT nanofibers. The increasing defect density thus leads to H-aggregation with an increasing degree of disorder along the nanofiber.

Evidence for the increasing disorder toward the P3HT nanofiber ends comes from the PL spectra at the different spots (Figure 2b, dashed lines). All PL spectra feature a distorted vibronic progression with a partially suppressed highest-energy peak around 1.9 eV, the electronic 0–0 transition, relative to the lower energy 0–1 transition around 1.7 eV. This spectral shape is typical for PL from H-aggregated P3HT.^46^ The absence of PL signal from the amorphous part above 2.0 eV is expected due to the relatively low absorbance of amorphous P3HT at the excitation wavelength (532 nm, corresponding to 2.33 eV). Moreover, rapid and efficient energy transfer from amorphous toward aggregated regions may occur prior to PL.^47,48^ The PL spectra exhibit systematic variations as a function of position, in particular, the relative intensity of the 0–0 peak changes, which implies different degrees of disorder.^46^ The increasing trend of the relative 0–0 PL intensity from spot A toward spot B demonstrates increasing disorder along P3HT nanofibers, corroborating our interpretation of the changes in absorption spectra. Notably, the spectral shape of the PL spectra with the suppressed 0–0 PL peaks highlights that the emission always stems from (vibronic) exciton states delocalized along the π-stacking direction, despite an increasing defect density toward nanofiber ends. In other words, such defects do not introduce (emissive and highly localized) trap states that modify emission properties; they rather modulate the overall excited-state energy landscape of delocalized vibronic excitons.

The spectral shift of the PL spectra as a function of position shows the opposite trend compared to the shift observed in absorption. Since some reabsorption may occur, we retrieve spectral shifts by extracting the spectral position *E*_01_^PL^ of the 0–1 PL peak with a simple peak tracking algorithm (SI, Section S4). The position of the 0–0 transition is then determined via *E*_00_^PL^ = *E*_01_^PL^ + *E*_vib_, with *E*_vib_ = 0.18 eV being the energy of the dominant carbon-bond stretch vibration coupling to the electronic transition.^49^ We find that the 0–0 PL peak position *E*_00_^PL^ red-shifts by ca. 40 meV starting from 1.90 eV at spot A (see Table 1). This opposite trend in spectral shifts between absorption and PL indicates complex structural and electronic relaxation processes related to the H-aggregation of P3HT that will be discussed in detail further below.

**Figure 3:**
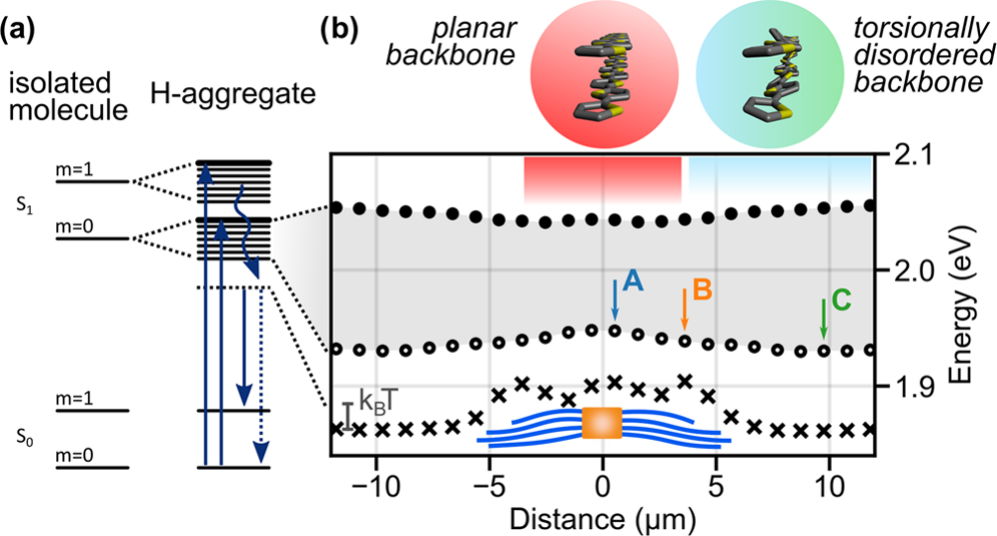
Excited-state energy landscape along P3HT nanofibers within a NA/P3HT superstructure. (a) Energy level schemes of isolated (left) and H-aggregated (right) molecules. Arrows indicate absorption, relaxation, and emission, and *m* represents the vibrational quantum number. (b) Excited-state energy landscape along the dashed white arrow in Figure 2a retrieved from optical spectra. The gray shaded area indicates the lowest-energy (*m* = 0) vibronic exciton band determined from spatially resolved absorption spectra. Filled and open circles indicate the upper and lower band edge, respectively. Crosses indicate the torsionally relaxed, lowest-energy exciton state from where emission occurs. The red and blue-green-filled circles and boxes (top) highlight the suggested planar and increasingly torsionally disordered P3HT backbones within P3HT nanofibers and into the surrounding P3HT film. The blue, orange, and green labels (A, B, C) refer to the same positions marked in Figure 2a.

To visualize the continuous variation in the excited-state energy landscape along our P3HT nanofiber, we performed the analysis outlined above for all 40 absorption and PL spectra along the dashed arrow in Figure 2a. For illustration, Figure 3a schematically shows the vibronic exciton bands of an H-aggregate (right) and how those relate to the corresponding energy levels of isolated noninteracting molecules (left) in the case of a single effective vibrational mode (here: carbon-bond stretch) coupling to the electronic transition.^4,20,44,50^ We limit ourselves to the bands with vibrational quantum numbers *m* = 0 and 1 for clarity. In an H-aggregate, only the top state of each band carries oscillator strength.^44^ Hence, the lowest-energy peak position *E*_A1_^Abs^ of the absorption spectra corresponds to the upper band edge of the lowest-energy (*m* = 0) vibronic exciton band. This upper band edge, determined from 24 spectra around the central supramolecular NA, is displayed as a function of the position along P3HT nanofibers in Figure 3b by filled circles, with the supramolecular NA being located at 0 μm. We find a minimum in the energy position of the upper band edge at the beginning of the P3HT nanofibers at the nucleating agent, a continuous increase (blue shift) toward the nanofibers’ ends, and finally, a leveling off to a constant value within the surrounding P3HT film.

The full shape of the (*m* = 0) vibronic exciton band is obtained by calculating the energy position of its lower band edge via subtraction of the bandwidth *W*_0_ from the energy of the upper band edge. *W*_0_ is related to the free exciton bandwidth W determined above via *W*_0_ = *W* * exp(*−S*).^44,46^*S* refers to the Huang–Rhys factor for noninteracting P3HT chains, which we determined previously by single-molecule spectroscopy to be *S* = 0.7^30,51^ (see also the SI, Section S3). This lower band edge (labeled with open circles in Figure 3b) has its highest energy at the beginning of the P3HT nanofibers, decreases in energy (red-shifts) along the nanofibers, and again levels off to a constant energy within the P3HT film. Hence, an energy gradient is imprinted in the bottom of the lowest-energy (*m* = 0) vibronic exciton band along P3HT nanofibers, which amounts to about thermal energy at room temperatures.

The spatially resolved PL spectra along the P3HT nanofibers show a red shift of the electronic 0–0 PL peak *E*_00_^PL^ (Figure 3b, crosses) similar to that of the lower band edge *E*_00_^Abs^ determined from absorption data. Yet, the behavior of the red shift at the position between the nanofiber region and the surrounding film is much steeper, as seen in the strong change of *E*_00_^PL^ at around ±5 μm, close to the P3HT nanofibers’ end. In this context, it is instructive to look at the energy difference between *E*_00_^PL^ and *E*_00_^Abs^. In the region of the P3HT nanofibers, we find an energy difference of about 20 meV, which significantly increases to 50 meV in the surrounding P3HT film due to the more pronounced change in *E*_00_^PL^ at the end of the nanofibers. Two effects can be responsible for this energy gap. First, energy transfer between crystalline domains within the P3HT nanofibers or the surrounding semicrystalline film can cause the emission to originate from crystallites with energetically very low-lying excited states. Such low-energy emitting crystallites, however, should be preferentially found close to the supramolecular NA at the beginning of the P3HT nanofibers. At that position, the relative 0–0 PL intensity is lowest (see Figure 2b), and thus the P3HT nanofibers possess a high degree of order with a larger intra-chain delocalization of electronic excitations, as discussed above. Since this is in contrast to our observation of higher energy emission at the beginning of P3HT nanofibers, we believe that this energy transfer cannot be the main effect. Second, structural relaxation, e.g., along the configuration coordinate of torsional modes of the P3HT backbone (planarization), can occur prior to the emission process. Such torsional relaxation takes place within picoseconds and was observed in P3HT aggregates.^33,52−54^ Our data thus suggest that torsional relaxation, after absorption and intra-band relaxation, becomes more and more pronounced when going along the P3HT nanofibers. In other words, the torsional disorder is smallest (chain planarity is highest) at the beginning of the nanofibers, and the disorder increases (planarity decreases) along the nanofiber growth direction (see the illustration in Figure 3b, top).

Based on our hyperspectral data set, the origin of the excited-state energy gradient along the growth direction of the P3HT nanofibers can thus be traced back to a decreasing degree of order caused by fractionation during polymer crystallization into nanofibers. During crystallization, an increasing number of defects are incorporated into P3HT nanofibers. Both regio and chain-end defects can give rise to an increasing torsional disorder of P3HT chains due to steric hindrance of hexyl side chains of neighboring chains within a crystalline domain. Since this increasing torsional disorder along the P3HT nanofibers limits intra-chain delocalization of electronic excitations, the electronic Coulomb interaction between π-stacked P3HT backbones (and thus the exciton bandwidth) increases along the nanofibers’ growth direction (see Figure 3b, gray shaded area). Ultimately, fractionation imprints a gradient into the excited-state energy landscape of P3HT nanofibers grown under controlled conditions. We note that in the surrounding P3HT film, comprising randomly oriented crystalline nanostructures, we do not find variations in the energy landscape, thus providing a direct control experiment for our approach.

**Figure 4:**
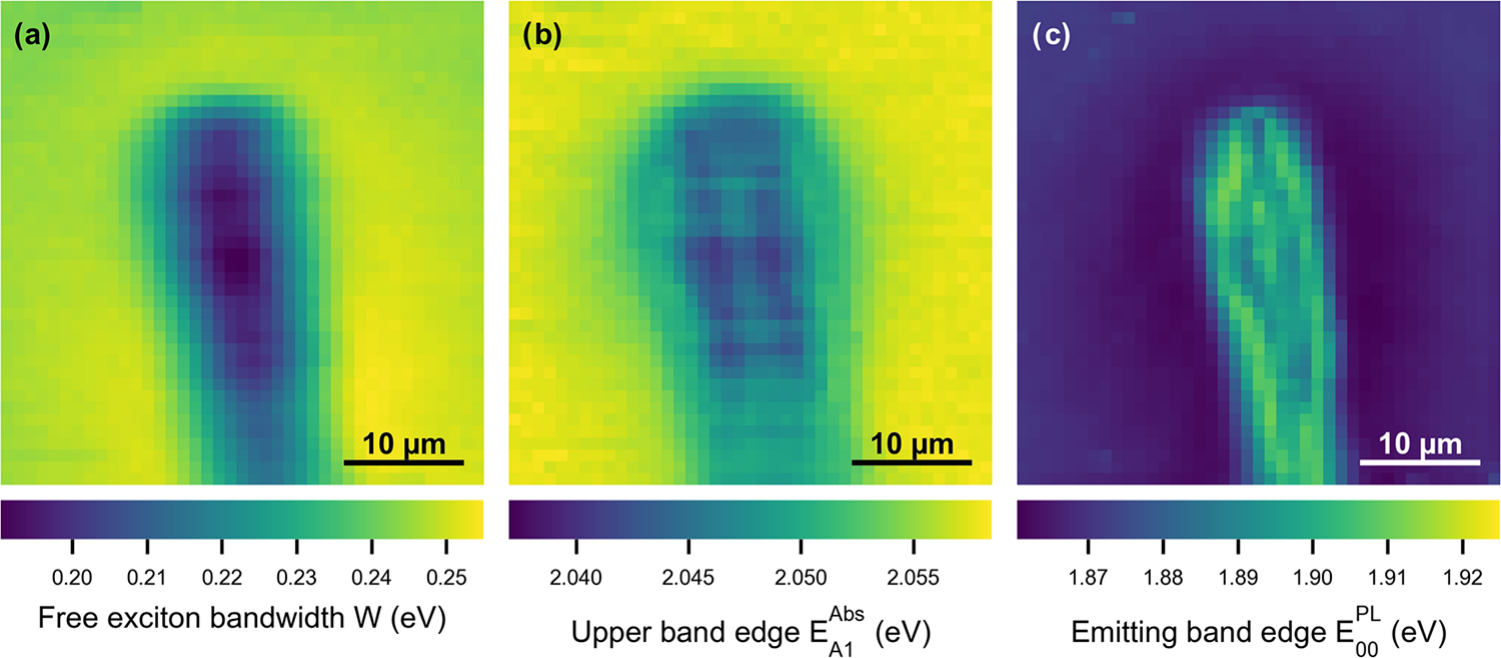
Hyperspectral maps of the NA/P3HT superstructure based on 1680 individual spectra. (a) Free exciton bandwidth *W*. (b) Upper band edge *E*_A1_^Abs^. (c) Emitting band edge *E*_00_^PL^. The 40 × 40 μm^2^ area is identical to that shown in Figures 1a and 2a.

The intrinsic energy gradient *along* the growth direction of P3HT nanofibers is most clearly seen in the lower band edge of the lowest-energy (*m* = 0) vibronic exciton band. Importantly, we observe this gradient not only for the specific “horizontal” direction along the dashed arrow in Figure 2a. Analyzing our full hyperspectral data set of the 40 × 40 μm^2^ area results in 2-dimensional maps of the parameters *W* (free exciton bandwidth), *E*_A1_^Abs^ (upper band edge), and *E*_00_^PL^ (emitting band edge). These 2-dimensional maps, shown in Figure 4 (see also the energy landscape cross sections in Figure S8), demonstrate that the presence of the observed gradients is not limited to one specific “horizontal” line, but gradients are imprinted consistently along the P3HT nanofibers’ growth direction, independent of the starting position at the supramolecular NA. In particular, small imperfections in the (otherwise rod-like) supramolecular NA, as visible in Figure 1a, do not significantly affect the formation of a defined gradient in the excited-state energy landscape. The robustness and reproducibility of our concept are confirmed by hyperspectral measurements in another NA/P3HT superstructure (see the SI, Figure S9).

## Conclusions

Creating a gradient in the excited-state energy landscape along well-defined supramolecular nanostructures is an intriguing approach toward steering exciton diffusion. To create such gradients, a variation of (photophysical) parameters along nanostructures is required, for instance, a change in stacking distance, in intermolecular electronic interaction, and/or in (electronic and structural) order. In supramolecular nano-objects based on conjugated polymers with broad molecular weight distribution including regio-defects, such intrinsic variation is induced by fractionation during polymer crystallization. We demonstrated this concept of a gradient in the excited-state landscape on a model system that comprises nanofibers of the prototypical conjugated polymer P3HT as part of a supramolecular superstructure. Exploiting an efficient supramolecular NA, P3HT nanofiber growth starts from a well-defined position, and P3HT nanofibers are highly oriented with lengths of several μm. This unique superstructure geometry is a prerequisite to allow investigations by hyperspectral optical imaging. Fractionation during the crystallization of P3HT incorporates an increasing number of defects (regio-defects, chain-end defects, and torsionally disordered chains) toward nanofiber ends. This leads to an increase in electronic inter-chain interaction of π-stacked P3HT chains along nanofibers. Thus, a “downhill” gradient of more than thermal energy at room temperature is imprinted in the bottom of the exciton band along the P3HT nanofibers. We emphasize that regio-defects, chain ends, and (intra-chain) torsional disorder do not introduce (emissive and strongly localized) traps for electronic excitations. The presence of such defects rather modulates the overall excited-state energy landscape of delocalized vibronic excitons to create an energy gradient over distances of several micrometers.

In principle, intrinsic energy gradients in the lowest-energy exciton states could promote directed energy transport along the nanofibers toward their ends, i.e., toward lower energies, because relaxed excitons are responsible for long-range transport.^55^ The absence of directed long-range transport in our P3HT nanofibers might be related to the relatively small crystalline domain size of only about 10 nm, which introduces inter-crystallite disorder and is detrimental to transport. However, we believe that our concept of fractionation-induced excited-state energy gradients is transferable to other combinations of nucleating agents and conjugated polymers that crystallize into fibrillar (nano-)structures. This approach may thus pave the way to achieving directed long-range energy transport and may find use in novel photonic nanodevices or as antennae for the guided transport of excitation energy in artificial light-harvesting systems.

## Supporting Information Available

Sample preparation and reference experiments, experimental section, structural characterization of P3HT nanofibers, analysis of spectroscopic data, and hyperspectral images (PDF)

## References

[ref1] Brédas J.-L.; Sargent E. H.; Scholes G. D. Photovoltaic Concepts Inspired by Coherence Effects in Photosynthetic Systems. Nat. Mater. 2017, 16, 35–44. 10.1038/nmat4767. 27994245

[ref2] Strümpfer J.; Şener M.; Schulten K. How Quantum Coherence Assists Photosynthetic Light-Harvesting. J. Phys. Chem. Lett. 2012, 3, 536–542. 10.1021/jz201459c. 22844553 PMC3404497

[ref3] Cogdell R. J.; Gall A.; Köhler J. The Architecture and Function of the Light-Harvesting Apparatus of Purple Bacteria: From Single Molecules to *in Vivo* Membranes. Q. Rev. Biophys. 2006, 39, 227–324. 10.1017/S0033583506004434. 17038210

[ref4] Brixner T.; Hildner R.; Köhler J.; Lambert C.; Würthner F. Exciton Transport in Molecular Aggregates - From Natural Antennas to Synthetic Chromophore Systems. Adv. Energy Mater. 2017, 7, 1700236 10.1002/aenm.201700236.

[ref5] Scholes G. D.; Mirkovic T.; Turner D. B.; Fassioli F.; Buchleitner A. Solar Light Harvesting by Energy Transfer: From Ecology to Coherence. Energy Environ. Sci. 2012, 5, 9374 10.1039/c2ee23013e.

[ref6] Kim J.; McQuade D. T.; Rose A.; Zhu Z.; Swager T. M. Directing Energy Transfer within Conjugated Polymer Thin Films. J. Am. Chem. Soc. 2001, 123, 11488–11489. 10.1021/ja016693g. 11707131

[ref7] Berggren M.; Dodabalapur A.; Slusher R. E.; Bao Z. Light Amplification in Organic Thin Films Using Cascade Energy Transfer. Nature 1997, 389, 466–469. 10.1038/38979.

[ref8] Sánchez-Mosteiro G.; van Dijk E. M. H. P.; Hernando J.; Heilemann M.; Tinnefeld P.; Sauer M.; Koberlin F.; Patting M.; Wahl M.; Erdmann R.; van Hulst N. F.; García-Parajó M. F. DNA-Based Molecular Wires: Multiple Emission Pathways of Individual Constructs. J. Phys. Chem. B 2006, 110, 26349–26353. 10.1021/jp064701f. 17181294

[ref9] Ajayaghosh A.; Praveen V. K.; Vijayakumar C.; George S. J. Molecular Wire Encapsulated into π Organogels: Efficient Supramolecular Light-Harvesting Antennae with Color-Tunable Emission. Angew. Chem., Int. Ed. 2007, 46, 6260–6265. 10.1002/anie.200701925. 17607676

[ref10] Rao K. V.; Datta K. K. R.; Eswaramoorthy M.; George S. J. Light-Harvesting Hybrid Hydrogels: Energy-Transfer-Induced Amplified Fluorescence in Noncovalently Assembled Chromophore-Organoclay Composites. Angew. Chem., Int. Ed. 2011, 50, 1179–1184. 10.1002/anie.201006270. 21268222

[ref11] Li C.; Zhang J.; Zhang S.; Zhao Y. Efficient Light-Harvesting Systems with Tunable Emission through Controlled Precipitation in Confined Nanospace. Angew. Chem., Int. Ed. 2019, 58, 1643–1647. 10.1002/anie.201812146. 30418700

[ref12] Liu Y.; Jin J.; Deng H.; Li K.; Zheng Y.; Yu C.; Zhou Y. Protein-Framed Multi-Porphyrin Micelles for a Hybrid Natural-Artificial Light-Harvesting Nanosystem. Angew. Chem., Int. Ed. 2016, 55, 7952–7957. 10.1002/anie.201601516. 27187799

[ref13] Zhang X.; Rehm S.; Safont-Sempere M. M.; Würthner F. Vesicular Perylene Dye Nanocapsules as Supramolecular Fluorescent PH Sensor Systems. Nat. Chem. 2009, 1, 623–629. 10.1038/nchem.368. 21378954

[ref14] Calver C. F.; Schanze K. S.; Cosa G. Biomimetic Light-Harvesting Antenna Based on the Self-Assembly of Conjugated Polyelectrolytes Embedded within Lipid Membranes. ACS Nano 2016, 10, 10598–10605. 10.1021/acsnano.6b07111. 27934088

[ref15] Acharyya K.; Bhattacharyya S.; Lu S.; Sun Y.; Mukherjee P. S.; Stang P. J. Emissive Platinum(II) Macrocycles as Tunable Cascade Energy Transfer Scaffolds. Angew. Chem., Int. Ed. 2022, 61, e202200715 10.1002/anie.202200715. 35107874

[ref16] Bösch C. D.; Langenegger S. M.; Häner R. Light-Harvesting Nanotubes Formed by Supramolecular Assembly of Aromatic Oligophosphates. Angew. Chem., Int. Ed. 2016, 55, 9961–9964. 10.1002/anie.201604508. 27375116

[ref17] Winiger C. B.; Li S.; Kumar G. R.; Langenegger S. M.; Häner R. Long-Distance Electronic Energy Transfer in Light-Harvesting Supramolecular Polymers. Angew. Chem., Int. Ed. 2014, 53, 13609–13613. 10.1002/anie.201407968. 25345576

[ref18] Winiger C. B.; Langenegger S. M.; Calzaferri G.; Häner R. Formation of Two Homo-Chromophoric H-Aggregates in DNA-Assembled Alternating Dye Stacks. Angew. Chem., Int. Ed. 2015, 54, 3643–3647. 10.1002/anie.201410041. 25649794

[ref19] Das G.; Cherumukkil S.; Padmakumar A.; Banakar V. B.; Praveen V. K.; Ajayaghosh A. Tweaking a BODIPY Spherical Self-Assembly to 2D Supramolecular Polymers Facilitates Excited-State Cascade Energy Transfer. Angew. Chem., Int. Ed. 2021, 60, 7851–7859. 10.1002/anie.202015390. 33427346

[ref20] Kreger K.; Schmidt H.-W.; Hildner R. Tailoring the Excited-State Energy Landscape in Supramolecular Nanostructures. Electron. Struct. 2021, 3, 023001 10.1088/2516-1075/abf485.

[ref21] Kim Y.; Cook S.; Tuladhar S. M.; Choulis S. A.; Nelson J.; Durrant J. R.; Bradley D. D. C.; Giles M.; McCulloch I.; Ha C.-S.; Ree M. A Strong Regioregularity Effect in Self-Organizing Conjugated Polymer Films and High-Efficiency Polythiophene:Fullerene Solar Cells. Nat. Mater. 2006, 5, 197–203. 10.1038/nmat1574.

[ref22] Adachi T.; Brazard J.; Ono R. J.; Hanson B.; Traub M. C.; Wu Z.-Q.; Li Z.; Bolinger J. C.; Ganesan V.; Bielawski C. W.; Vanden Bout D. A.; Barbara P. F. Regioregularity and Single Polythiophene Chain Conformation. J. Phys. Chem. Lett. 2011, 2, 1400–1404. 10.1021/jz200546x.

[ref23] Tatum W. K.; Resing A. B.; Flagg L. Q.; Ginger D. S.; Luscombe C. K. Defect Tolerance of π-Conjugated Polymer Crystal Lattices and Their Relevance to Optoelectronic Applications. ACS Appl. Polym. Mater. 2019, 1, 1466–1475. 10.1021/acsapm.9b00223.

[ref24] Roehling J. D.; Arslan I.; Moulé A. J. Controlling Microstructure in Poly(3-Hexylthiophene) Nanofibers. J. Mater. Chem. 2012, 22, 2498–2506. 10.1039/C2JM13633C.

[ref25] Oosterbaan W. D.; Vrindts V.; Berson S.; Guillerez S.; Douhéret O.; Ruttens B.; D’Haen J.; Adriaensens P.; Manca J.; Lutsen L.; Vanderzande D. Efficient Formation, Isolation and Characterization of Poly(3-Alkylthiophene) Nanofibres: Probing Order as a Function of Side-Chain Length. J. Mater. Chem. 2009, 19, 5424 10.1039/b900670b.

[ref26] Wenzel F. A.; Welz H.; van der Zwan K. P.; Stäter S.; Kreger K.; Hildner R.; Senker J.; Schmidt H.-W. Highly Efficient Supramolecular Nucleating Agents for Poly(3-Hexylthiophene). Macromolecules 2022, 55, 2861–2871. 10.1021/acs.macromol.1c02283.

[ref27] Brinkmann M. Structure and Morphology Control in Thin Films of Regioregular Poly(3-Hexylthiophene). J. Polym. Sci., Part B: Polym. Phys. 2011, 49, 1218–1233. 10.1002/polb.22310.

[ref28] Noriega R.; Rivnay J.; Vandewal K.; Koch F. P. V.; Stingelin N.; Smith P.; Toney M. F.; Salleo A. A General Relationship between Disorder, Aggregation and Charge Transport in Conjugated Polymers. Nat. Mater. 2013, 12, 1038–1044. 10.1038/nmat3722. 23913173

[ref29] Beer P.; Reichstein P. M.; Schötz K.; Raithel D.; Thelakkat M.; Köhler J.; Panzer F.; Hildner R. Disorder in P3HT Nanoparticles Probed by Optical Spectroscopy on P3HT-b-PEG Micelles. J. Phys. Chem. A 2021, 125, 10165–10173. 10.1021/acs.jpca.1c08377. 34797986 PMC8647091

[ref30] Raithel D.; Baderschneider S.; de Queiroz T. B.; Lohwasser R.; Köhler J.; Thelakkat M.; Kümmel S.; Hildner R. Emitting Species of Poly(3-Hexylthiophene): From Single, Isolated Chains to Bulk. Macromolecules 2016, 49, 9553–9560. 10.1021/acs.macromol.6b02077.

[ref31] Scharsich C.; Lohwasser R. H.; Sommer M.; Asawapirom U.; Scherf U.; Thelakkat M.; Neher D.; Köhler A. Control of Aggregate Formation in Poly(3-Hexylthiophene) by Solvent, Molecular Weight, and Synthetic Method. J. Polym. Sci., Part B: Polym. Phys. 2012, 50, 442–453. 10.1002/polb.23022.

[ref32] Panzer F.; Bässler H.; Lohwasser R.; Thelakkat M.; Köhler A. The Impact of Polydispersity and Molecular Weight on the Order–Disorder Transition in Poly(3-Hexylthiophene). J. Phys. Chem. Lett. 2014, 5, 2742–2747. 10.1021/jz5009938. 26277973

[ref33] Banerji N.; Cowan S.; Vauthey E.; Heeger A. J. Ultrafast Relaxation of the Poly(3-Hexylthiophene) Emission Spectrum. J. Phys. Chem. C 2011, 115, 9726–9739. 10.1021/jp1119348.

[ref34] Brinkmann M.; Chandezon F.; Pansu R. B.; Julien-Rabant C. Epitaxial Growth of Highly Oriented Fibers of Semiconducting Polymers with a Shish-Kebab-Like Superstructure. Adv. Funct. Mater. 2009, 19, 2759–2766. 10.1002/adfm.200900966.

[ref35] Agbolaghi S.; Charoughchi S.; Aghapour S.; Abbasi F.; Bahadori A.; Sarvari R. Bulk Heterojunction Photovoltaics with Improved Efficiencies Using Stem-Leaf, Shish-Kebab and Double-Fibrillar Nano-Hybrids Based on Modified Carbon Nanotubes and Poly(3-Hexylthiophene). Sol. Energy 2018, 170, 138–150. 10.1016/j.solener.2018.05.064.

[ref36] Liu J.; Zou J.; Zhai L. Bottom-up Assembly of Poly(3-Hexylthiophene) on Carbon Nanotubes: 2D Building Blocks for Nanoscale Circuits. Macromol. Rapid Commun. 2009, 30, 1387–1391. 10.1002/marc.200900225. 21638395

[ref37] Bu L.; Pentzer E.; Bokel F. A.; Emrick T.; Hayward R. C. Growth of Polythiophene/Perylene Tetracarboxydiimide Donor/Acceptor Shish-Kebab Nanostructures by Coupled Crystal Modification. ACS Nano 2012, 6, 10924–10929. 10.1021/nn3043836. 23163922

[ref38] Samitsu S.; Shimomura T.; Heike S.; Hashizume T.; Ito K. Effective Production of Poly(3-Alkylthiophene) Nanofibers by Means of Whisker Method Using Anisole Solvent: Structural, Optical, and Electrical Properties. Macromolecules 2008, 41, 8000–8010. 10.1021/ma801128v.

[ref39] Brinkmann M.; Rannou P. Effect of Molecular Weight on the Structure and Morphology of Oriented Thin Films of Regioregular Poly(3-Hexylthiophene) Grown by Directional Epitaxial Solidification. Adv. Funct. Mater. 2007, 17, 101–108. 10.1002/adfm.200600673.

[ref40] Liu J.; Arif M.; Zou J.; Khondaker S. I.; Zhai L. Controlling Poly(3-Hexylthiophene) Crystal Dimension: Nanowhiskers and Nanoribbons. Macromolecules 2009, 42, 9390–9393. 10.1021/ma901955c.

[ref41] Wu Z.; Petzold A.; Henze T.; Thurn-Albrecht T.; Lohwasser R. H.; Sommer M.; Thelakkat M. Temperature and Molecular Weight Dependent Hierarchical Equilibrium Structures in Semiconducting Poly(3-Hexylthiophene). Macromolecules 2010, 43, 4646–4653. 10.1021/ma902566h.

[ref42] Ye G.; Liu J.; Qiu X.; Stäter S.; Qiu L.; Liu Y.; Yang X.; Hildner R.; Koster L. J. A.; Chiechi R. C. Controlling N-Type Molecular Doping via Regiochemistry and Polarity of Pendant Groups on Low Band Gap Donor–Acceptor Copolymers. Macromolecules 2021, 54, 3886–3896. 10.1021/acs.macromol.1c00317. 34054145 PMC8154869

[ref43] Clark J.; Chang J.-F.; Spano F. C.; Friend R. H.; Silva C. Determining Exciton Bandwidth and Film Microstructure in Polythiophene Films Using Linear Absorption Spectroscopy. Appl. Phys. Lett. 2009, 94, 163306 10.1063/1.3110904.

[ref44] Spano F. C. The Spectral Signatures of Frenkel Polarons in H- and J-Aggregates. Acc. Chem. Res. 2010, 43, 429–439. 10.1021/ar900233v. 20014774

[ref45] Gierschner J.; Huang Y.-S.; Van Averbeke B.; Cornil J.; Friend R. H.; Beljonne D. Excitonic versus Electronic Couplings in Molecular Assemblies: The Importance of Non-Nearest Neighbor Interactions. J. Chem. Phys. 2009, 130, 044105 10.1063/1.3065267. 19191375

[ref46] Spano F. C. Modeling Disorder in Polymer Aggregates: The Optical Spectroscopy of Regioregular Poly(3-Hexylthiophene) Thin Films. J. Chem. Phys. 2005, 122, 234701 10.1063/1.1914768. 16008467

[ref47] Reichenberger M.; Baderschneider S.; Kroh D.; Grauf S.; Köhler J.; Hildner R.; Köhler A. Watching Paint Dry: The Impact of Diiodooctane on the Kinetics of Aggregate Formation in Thin Films of Poly(3-Hexylthiophene). Macromolecules 2016, 49, 6420–6430. 10.1021/acs.macromol.6b01257.

[ref48] Shaw P. E.; Ruseckas A.; Samuel I. D. W. Exciton Diffusion Measurements in Poly(3-Hexylthiophene). Adv. Mater. 2008, 20, 3516–3520. 10.1002/adma.200800982.

[ref49] Louarn G.; Trznadel M.; Buisson J. P.; Laska J.; Pron A.; Lapkowski M.; Lefrant S. Raman Spectroscopic Studies of Regioregular Poly(3-Alkylthiophenes). J. Phys. Chem. A 1996, 100, 12532–12539. 10.1021/jp960104p.

[ref50] Spano F. C.; Silva C. H- and J-Aggregate Behavior in Polymeric Semiconductors. Annu. Rev. Phys. Chem. 2014, 65, 477–500. 10.1146/annurev-physchem-040513-103639. 24423378

[ref51] Raithel D.; Simine L.; Pickel S.; Schötz K.; Panzer F.; Baderschneider S.; Schiefer D.; Lohwasser R.; Köhler J.; Thelakkat M.; Sommer M.; Köhler A.; Rossky P. J.; Hildner R. Direct Observation of Backbone Planarization via Side-Chain Alignment in Single Bulky-Substituted Polythiophenes. Proc. Natl. Acad. Sci. U.S.A. 2018, 115, 2699–2704. 10.1073/pnas.1719303115. 29483262 PMC5856543

[ref52] Westenhoff S.; Beenken W. J. D.; Friend R. H.; Greenham N. C.; Yartsev A.; Sundström V. Anomalous Energy Transfer Dynamics Due to Torsional Relaxation in a Conjugated Polymer. Phys. Rev. Lett. 2006, 97, 166804 10.1103/PhysRevLett.97.166804. 17155424

[ref53] Parkinson P.; Müller C.; Stingelin N.; Johnston M. B.; Herz L. M. Role of Ultrafast Torsional Relaxation in the Emission from Polythiophene Aggregates. J. Phys. Chem. Lett. 2010, 1, 2788–2792. 10.1021/jz101026g.

[ref54] Gallaher J. K.; Chen K.; Huff G. S.; Prasad S. K. K.; Gordon K. C.; Hodgkiss J. M. Evolution of Nonmirror Image Fluorescence Spectra in Conjugated Polymers and Oligomers. J. Phys. Chem. Lett. 2016, 7, 3307–3312. 10.1021/acs.jpclett.6b01185. 27485296

[ref55] Vlaming S. M.; Malyshev V. A.; Eisfeld A.; Knoester J. Subdiffusive Exciton Motion in Systems with Heavy-Tailed Disorder. J. Chem. Phys. 2013, 138, 214316 10.1063/1.4808155. 23758380

