# Supplementary material for: Directed Gradients in the Excited-State Energy Landscape of Poly(3-hexylthiophene) Nanofibers
Source: J Am Chem Soc. 2023 Jun 14;145(25):13780–7. doi: 10.1021/jacs.3c02117 (PMC10311527; doi:10.1021/jacs.3c02117)
Supplement: Supplementary file 1 — ja3c02117_si_001.pdf [file ja3c02117_si_001.pdf]

Supporting Information:

Directed Gradients in the Excited-State Energy Landscape of Poly(3-hexylthiophene) Nanofibres

[1, 2]Sebastian Stäter, [3]Felix A. Wenzel, [3]Hannes Welz, [3]Klaus Kreger,[2,4] Jürgen Köhler, [3]Hans-Werner Schmidt and [1,2]Richard Hildner*
[1] Zernike Institute for Advanced Materials, University of Groningen, Nijenborgh 4, 9747 AG Groningen, The Netherlands
[2] Spectroscopy of Soft Matter, University of Bayreuth, Universitätsstrasse 30, 95440 Bayreuth, Germany
[3] Macromolecular Chemistry I and Bavarian Polymer Institute, University of Bayreuth, Universitätsstrasse 30, 95440 Bayreuth, Germany
[4] Bavarian Polymer Institute and Bayreuther Institut für Makromolekülforschung (BIMF), University of Bayreuth, 95440 Bayreuth, Germany

S1: Materials and Methods....3
- Materials....3
- P3HT crystallization in solution....3
- Preparation of NA/P3HT supramolecular superstructures....4
- Experimental setup for spatially resolved spectroscopy....5
- Experimental details for Electron Microscopy and SAED....6

S2: Structural Characterization of P3HT Nanofibres....7
S3: Analysis of Absorption Spectra....10
S4: Analysis of PL Spectra....12
S5: Additional cross-sections....13
S6: Additional dataset....14
S7: References....15

S1: Materials and Methods

Materials

Poly(3-hexylthiophene), P3HT, with a molecular weight M_w = 57 kg/mol, a dispersity Đ = 2.4 as determined by size exclusion chromatography [1], and a regioregularity of >96% was purchased from Rieke and used as received. The synthesis and characterisation of compound **1** (N,N'-1,4-phenylenebis[4-pyridinecarboxamide]) was described in a recent publication [1]. Chlorobenzene was purchased from abcr (HPLC grade, 99.5%).

P3HT crystallization in solution

Recently, we highlighted the outstanding nucleation performance of the supramolecular nucleating agent (NA) consisting of N,N'-1,4-phenylenebis[4-pyridinecarboxamide] (compound **1**) in the P3HT melt [1]. To show its nucleating abilities *in solution*, we first performed temperature-dependent UV-VIS spectroscopy of 1wt% P3HT-chlorobenzene solutions with different amounts of compound **1** added. All solutions were slowly cooled from 90°C to -5°C (with a cooling rate of roughly 1 K/h), resulting in a reduction of the absorption signal in the range of 400-550 nm, where amorphous P3HT absorbs, and the emergence of spectral features around 550-650 nm, where crystalline P3HT absorbs[2]. Figure S1 shows the P3HT crystallization process by plotting the absorption of crystalline P3HT at 620nm in the temperature range from 16°C to -2°C. At elevated temperatures, the absorption at this wavelength is low because the P3HT is completely dissolved. Upon cooling, the absorbance at 620nm rises steeply due to crystallisation of P3HT. In the solution without NA (blue line), this steep increase (the crystallisation) occurs at temperatures below 3°C. The addition of the NA (at concentrations of 0.01wt%, 0.1wt%, or 1wt%, see dashed and dotted lines) increases the crystallization temperature to around 4°C, clearly indicating a nucleation effect. Therefore, the supramolecular NA based on compound **1** can be readily used to initialise the crystallisation of P3HT in solution.

We note that the dynamic nature of the measurement in figure S1 means that only crystallization processes over time intervals of minutes to hours can be detected reliably, a situation similar to dynamic DSC measurements. As shown in figure S2, nucleation at room temperature is possible as well, but requires several days of crystallization time.

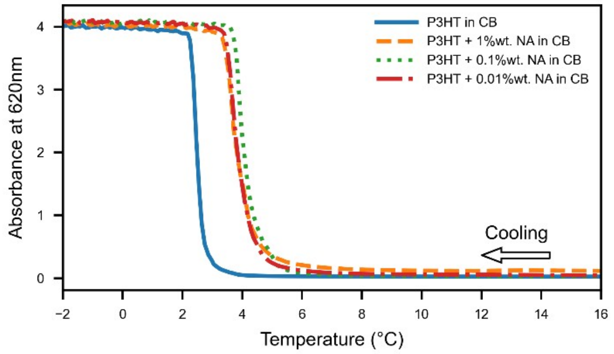

Figure S1: *Temperature-dependent absorption signal from P3HT-chlorobenzene (CB) solutions taken at a wavelength of 620 nm, corresponding to the absorption of crystalline P3HT without (blue) and with the addition of different amounts of compound **1** (orange: 1wt%, green: 0.1wt%, red: 0.01wt%). The presence of the supramolecular NA based on compound **1** increases the crystallization temperature, indicating nucleation of P3HT in the solution. All data were obtained while cooling the solutions from 90°C to -5°C.*

Preparation of NA/P3HT supramolecular superstructures

The P3HT nanofibres were grown as part of a supramolecular superstructure in a two-step protocol. At first, a solution of compound **1** in chlorobenzene at a concentration of 0.01wt% was heated to 130°C and cooled down to room temperature, resulting in the formation of ribbon-like supramolecular NA structures (**Figure S2a**). Subsequently, P3HT was added to the NA dispersion to reach a concentration of 1wt% (resulting in a concentration of 1wt% of compound **1** in relation to P3HT). The mixture was heated to 50°C, then cooled down to room temperature and aged for different times (main text: 11 days, figure S2c: 1 day, figure S9: 7 days). After ageing the superstructures were deposited on a glass coverslip by spin-coating.

Figure S2 demonstrates that the supramolecular NA enables the formation of the superstructures. Figure S2a shows a typical example of a SEM image of the ribbon-like supramolecular NA without the addition of P3HT, i.e., after the first step of the protocol. **Figure S2b** shows the SEM image of a thin film of P3HT without the addition of compound **1**. Only a grainy structure is observed, as expected for semicrystalline P3HT, and no defined structures can be found. Finally, NA/P3HT superstructures can be reliably formed by following the complete two-step protocol, as shown in **Figure S2c**. This demonstrates that the supramolecular NA is essential to achieve highly aligned, oriented and densely packed P3HT nanofibres as part of the NA/P3HT superstructures. In other words, the solvent chlorobenzene *requires* the use of the supramolecular NA to achieve nanofibre growth at room temperature.

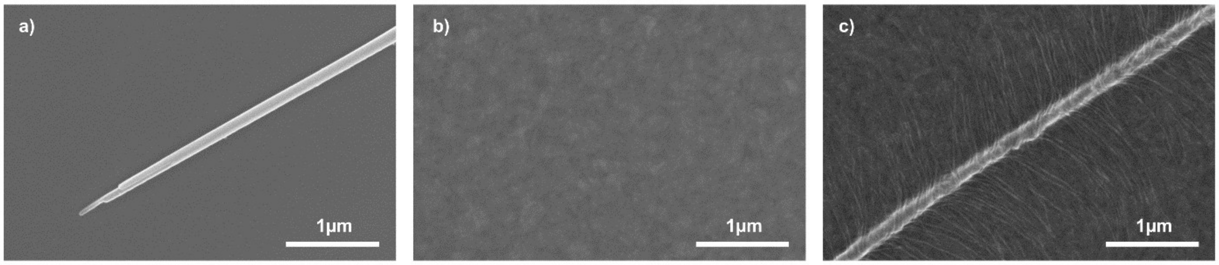

Figure S2: *Scanning electron microscopy images of (a) the supramolecular NA (without P3HT), (b) a semicrystalline P3HT film without compound **1**, and (c) the NA/P3HT superstructure prepared by the two-step process outlined in the text.*

Experimental setup for spatially resolved spectroscopy

For optical and spectroscopic experiments, 25µl of the NA/P3HT superstructure dispersion was spin-coated (Coros OP15, Siemens, with CONVAC 1001) onto glass slides at 1000rpm for 60s.

Optical imaging and spectroscopy were performed with a home-built inverted optical scanning microscope that can be operated in confocal and widefield mode. For PL measurements, the sample was excited with an unpolarized pulsed laser at 532 nm (NKT Fianium SC-400) through an oil-immersion objective (Olympus PlanApo, NA 1.45). The time-averaged excitation power was 40µW/cm^2 in widefield mode, and 1.5kW/cm^2 in confocal mode. Scanning was performed by moving the sample with a computer-controlled piezo stage (Piezosystem Jena Tritor 102SG). The PL signal was collected with the same objective and directed through a dichroic mirror (540nm, AHF) and a longpass filter (545nm, AHF) to suppress residual laser light. In confocal mode, the PL signal was focused onto the slit of an imaging spectrograph (Princeton Instruments IsoPlane 160, 150g/mm) and detected with a cooled emCCD camera (Andor iKon M DU934N-BR-DD, -80°C). In widefield mode, the PL signal was directed onto a sCMOS camera (pco PixelFly USB) for imaging. For brightfield microscopy and absorption spectroscopy, the sample was illuminated from above (trans-illumination) in widefield mode with a tungsten white light source (Streppel halolux 150), and filters were removed. Absorption spectra were calculated according to the Beer-Lambert-law, using reference spectra collected without a sample. The spectral resolution is limited to 0.4nm due to the pixel size of our detector. Based on the slit width of the spectrometer (100 µm), the microscope's magnification (67x), pixel size of emCCD, and hardware binning, the spatial resolution is 1µm by 1.6µm. The step size during sample scanning was, therefore, chosen to 1µm accordingly. We note that due to the nanoscopic dimensions of the investigated P3HT nanofibers (width ~20 nm), our optical experiments inevitably probe the average spectral response of several nanofibres as well as of along the nanofibres' growth direction.

Experimental details for Electron Microscopy and SAED

Scanning Electron Microscopy (SEM) measurements were performed with a Zeiss Ultra Plus (3kV, InLens detector) after sputter-coating the sample with a thin platinum layer. The relevant sample part containing a NA/P3HT superstructure was identified with an optical microscope (Zeiss Axio Imager.A2m) and transferred to the SEM using a universal sample holder for correlative optical and electron microscopy.

Selected area electron diffraction (SAED) was performed with a Zeiss / LEO EM922 Omega with an aperture diameter of 1 µm. The sample was prepared by filtering the NA/P3HT dispersion and depositing the redispersed filter cake onto a carbon-coated TEM copper grid. Finally, the samples were dried under vacuum.

S2: Structural Characterization of P3HT Nanofibres

To obtain structural information of P3HT nanofibres via TEM imaging and SAED, we deposited the final dispersion containing NA/P3HT superstructures, after filtration and redispersion, onto a carbon-coated TEM grid. In the first step, we located a NA/P3HT superstructure within the accessible squares of the grid, as shown in the overview image in **Figure S3**.

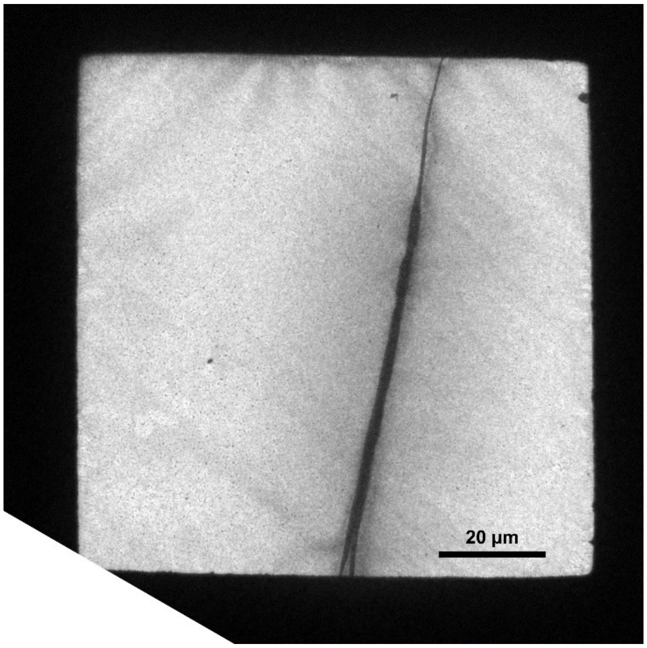

***Figure S3**: Overview TEM image of the investigated NA/P3HT superstructure. The supramolecular NA lies vertically in the image. The black frame is caused by the copper grid with 100µm wide openings.*

We then followed a systematic approach to perform both imaging and selected area electron diffraction along the P3HT nanofibres that extend from the supramolecular NA: Using a high magnification and moving the sample stepwise by around 1µm, we recorded 28 TEM images and SAED patterns along the P3HT nanofibers. We started at a position close to the supramolecular NA and moved towards the ends of the P3HT nanofibres. The aperture diameter for both imaging and SAED was 1µm.

Figures S4a and **c** are TEM images of an array of P3HT nanofibres located approximately 2µm and 7µm, respectively, from the supramolecular NA. As in Fig. 1b of the main text, the P3HT-fibers are oriented horizontally in the image, parallel with respect to each other, and show a homogeneous width.

Figure S4b and **d** show the SAED patterns of the same areas as shown in the TEM images. In general, this data agrees well with known diffraction data of P3HT nanofibres with edge-on

orientation [3–5]. The horizontal axis corresponds to the long fiber axis in real space, along which P3HT is stacked in a π-π-fashion. We identify the 020 and 040 diffraction signals of P3HT at scattering vectors q = 2.6 1/nm and q = 5.3 1/nm, with the 020 signal being the strongest signal observed. This corresponds to a stacking distance d = 0.38nm along the nanofibres' long axis. In the vertical direction, we identify the 002 signal at q = 2.6 1/nm. This corresponds to a distance d = 0.38nm along the axis defined by the conjugated P3HT backbones, which is the distance between thiophene monomers along a P3HT-chain. We note that separating the 020 and 002 signals would not be possible in isotropic samples, as both signals share the same q-value.

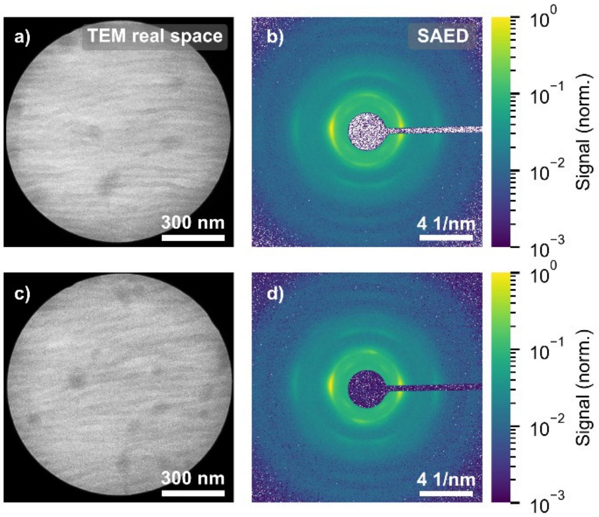

***Figure S4**: Structural analysis of P3HT nanofibres within a NA/P3HT superstructure. **(a, c)** TEM images of P3HT nanofibres with parallel alignment. **(b, d)** SAED patterns of the areas in (a, c). The two investigated spots were 2µm (a, b) and 7µm (c, d) away from the supramolecular NA.*

To rule out that a variation in the π-π-stacking distance along P3HT nanofibres causes the variation of the exciton bandwidth that we present in table 1 and figure 3 of the main text, we extract the position of the 020 peak for each of the 28 SAED patterns that we recorded systematically along the P3HT nanofibres. **Figure S5** shows that, along the P3HT nanofibres, the 020 peak appears in the range between 2.64/nm and 2.59/nm, corresponding to stacking distances d of 0.379nm and 0.387nm. In other words, the stacking distance d fluctuates by approximately 2%. Using the point-dipole-approximation with parallel transition dipole moments, the interaction energy V between two chromophores is given by $V=\frac{1}{4\pi\epsilon_0}\frac{p_1 p_2}{r^3}$, with p_1 and p_2 being the associated transition dipole moments, and r the distance between their centres of mass. For constant magnitudes of transition dipole moments, a 2% change of the π-π-stacking distance to $r_{long}=1.02\,r_{short}$ changes the interaction V by about 6%: $\frac{V_{long}}{V_{short}}=\frac{r_{short}^3}{r_{long}^3}=\frac{1}{1.02^3}=0.942$. In the main text however, we observe a decrease of the exciton bandwidth by up to 23%:

$\frac{193\,meV}{248\,meV} = 0.77$, which thus cannot be explained by the observed variation of the π-π-stacking distance. We further emphasise that, compared to the point-dipole-approximation, quantum chemical calculations on polythiophene stacks predict an even lower influence of the stacking distance on the interaction energy [6], so that our estimate represents an upper bound for the distance-related change in inter-chain interactions.

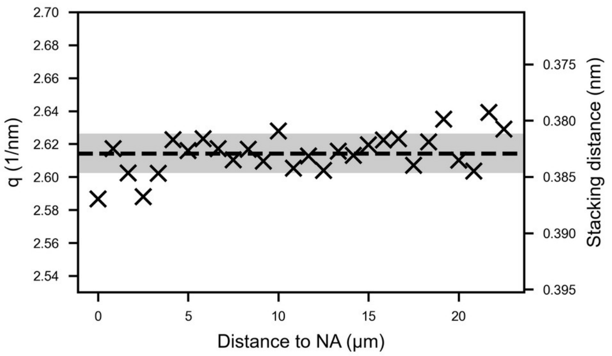

***Figure S5**: π-π-stacking distance along the long axis of P3HT nanofibres. Data points correspond to the centre of a Gaussian 2D fit to the 020 peak in SAED patterns. The black dashed line highlights the mean value, and the shaded area indicates the standard deviation.*

Our structural analysis thus confirms that the P3HT nanofibres are highly crystalline along their long axis with minimal structural variations, as evidenced by the diffraction pattern as a function of the spatial position.

S3: Analysis of Absorption Spectra

For the analysis of the absorption spectra of P3HT nanofibres, we use the Frenkel Polaron model that describes the optical properties of molecular H-aggregates in the presence of intra-molecular vibrations and that has been widely applied to conjugated polymers such as P3HT [7,8].We fit the low-energy part (1.87eV - 2.25 eV) of each absorption spectrum with equation (1), which models the energy-dependent absorption *A(E)* of crystalline P3HT as a distorted vibronic progression using a single (effective) vibrational mode:

$$\frac{A(E)}{E} \propto \sum_{m} \frac{S^m}{m!} \cdot \left(1 - \frac{W e^{-S}}{2 E_{vib}} G_m\right) \cdot \Gamma_m(E) \quad \text{with} \quad G_m = \sum_{n(\neq m)} \frac{S^n}{n!(n-m)} \qquad (1)$$

Here, m and n are vibrational quantum numbers, $\Gamma_m(E)$ is a Gaussian line-shape function centred around an energy E_m with $E_m = E^{Abs}_{A1} + m * E_{vib}$. The parameter E^{Abs}_{A1} describes the spectral position of the lowest-energy transition at around 2.1 eV, and E_{vib}=0.18 eV is the fixed energy of the (effective) vibrational mode (carbon-bond stretch) coupling to the electronic transition. The Huang-Rhys parameter S is a measure for the electron-phonon coupling strength and has to be taken from molecularly dissolved, non-interacting P3HT. Based on our recent single-molecule work, we keep the Huang-Rhys parameter constant at S=0.7 [9]. W is the free-exciton bandwidth and is related to the electronic interaction V between P3HT chains by $W=4V$ in the nearest-neighbour approximation. For W=0, the vibronic progression of the non-interacting P3HT is recovered, while for $W \geq 0$, the lowest-energy peak at 2.1 eV is reduced in relative intensity. Hence, W is the main fit parameter to describe the changes in the shape of the low-energy absorption.

Related to the choice of the Huang-Rhys parameter for our analysis we note that in Raithel et al. (2016) we reported S = 0.84 for a disordered P3HT film [9]. In films, however, inter-chain interactions are always present that lead to H-type coupling between chains and thus to an overestimation of the Huang-Rhys parameter via a suppression of the 0-0 PL peak relative to the 0-1 PL peak. In Raithel et al. (2018) we reported a value of S = 0.77 derived from single chains of a thiophene-derivative (PDOPT) with bulky side groups [10]. Single PDOPT chains are more planar than single P3HT chains and show a slightly higher electron-phonon coupling to effective carbon-bond stretch modes, and thus a slightly larger S-value. Hence, we chose S = 0.7 (unpublished results).

Figure S6 shows the best fits to the absorption spectra at positions A, B, and C (see Figure 2 of the main text). We fixed the linewidth of the lowest-energy transition (around 2.1 eV) at 80% of the (variable) overall linewidth for all transitions. This is justified since the vibronic transitions in bulk P3HT are, in fact, a superposition of several closely spaced individual vibrational modes, which gives rise to additional line broadening[9,10]. The residuals (difference between the data and best fit, green curves) nicely reproduce the absorption of amorphous P3HT with a maximum absorption slightly above 2.5 eV.

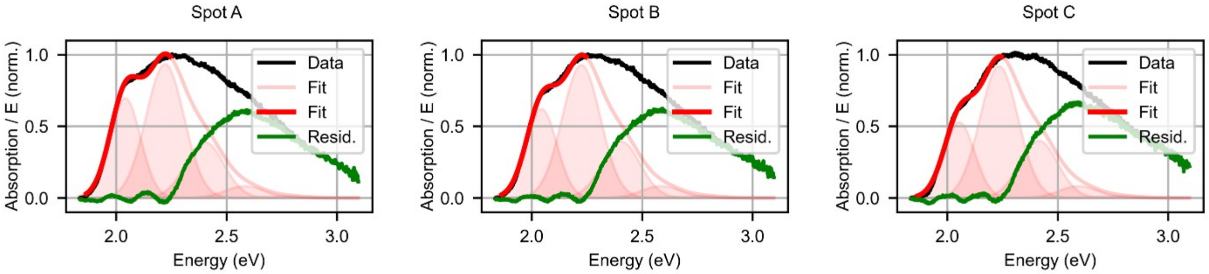

Figure S6: *Fitting of spatially resolved absorption spectra of P3HT nanofibres at spots A (left), B (centre), and C (right) from figure 2 of the main text. The black lines represent data, the red lines show the best fits to the low-energy region (1.87 eV - 2.25 eV), and the green lines are the residuals. The red-shaded areas represent the individual peaks of the vibronic progression. The residuals (green) in the high-energy region reproduce the absorption of amorphous P3HT.*

S4: Analysis of PL Spectra

PL spectra of crystalline P3HT can in principle be modelled with a distorted Franck-Condon progression[2,11]. Since our PL spectra may be affected by some reabsorption in the spectral region of the electronic 0-0 transition (1.9 to 2.0 eV), induced by a substantial optical density of around 1.0 of our sample, we refrain of a quantitative analysis of the shape of PL spectra. We note, however, that reabsorption has only a minor effect: E.g. the PL spectrum at spot C (Fig. S7) features the smallest relative intensity of the 0-0 PL peak; at the same time the corresponding absorption spectrum (Fig. S6) has the smallest absorbance in the spectral range where absorption overlaps with PL. This trend is inconsistent with reabsorption processes.

As only quantitative parameter we extract here the spectral shifts of the PL spectra as a function of position. We employ a simple peak tracking analysis. We fit the peak of the PL spectra (in the range 1.65eV to 1.80eV), which corresponds to the 0-1 transition E_{01}, with a parabola and extract its apex. **Figure S7** shows examples for this peak tracking for positions A, B, and C in Figure 2 of the main text. The position of the 0-0 PL peak is then calculated by $E^{PL}_{00} = E_{01} - E_{vib}$, with E_{vib} = 0.18 eV, see section S3.

For both the analysis of absorption and PL spectra, the employed Levenberg-Marquard fitting algorithm, directly yields the estimated standard-error of the best-fit values. For the values reported in Table 1 and figure 3 of the main text, these errors are in the range of 0.0004 eV (for E^{Abs}_{00} and E^{PL}_{00}) and 1 meV (for W). Because these errors are smaller than the symbols in figure 3, we refrain from showing error bars.

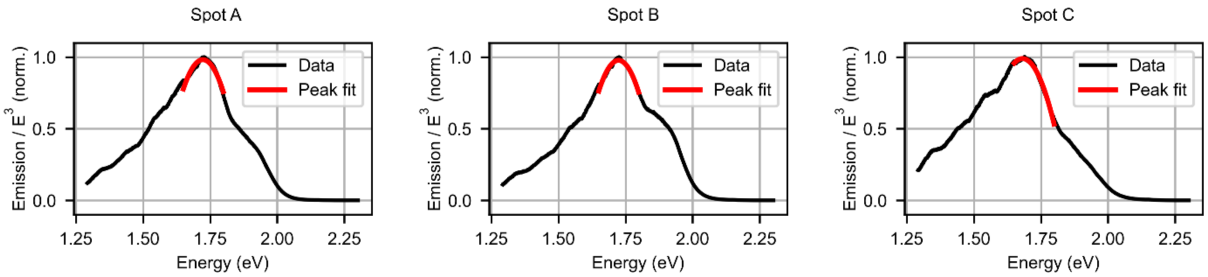

Figure S7: *Tracking of the 0-1 PL peak in spatially resolved PL spectra of P3HT nanofibres. The data correspond to the spots A (left), B (centre), and C (right) from figure 2 of the main text.*

S5: Additional energy landscape cross-sections

Analyzing the hyperspectral dataset, which covers an area of 40x40 µm² (and thus the whole area shown in figures 1a and 2a of the main text), allows us to extract the excited-state energy landscape at arbitrary positions along the direction of the supramolecular NA (perpendicularly to the P3HT nanofibres).

In analogy to Figure 3 of the main text, **Figure S8** shows the extracted shape of the *m*=0 vibronic exciton band (circles) and the position E_{PL}^{00} of the 0-0 PL peak (crosses) along additional horizontal axes, marked with the colored lines A (blue), B (orange, corresponds to the axis chosen in the main text and shown in Figure 3), and C (green) in Figure S8a. The corresponding excited-state energy landscapes, shown in Figure S8b, are essentially identical, demonstrating that the observed energy gradients are imprinted consistently into our NA/P3HT superstructure. Note that the NA/P3HT superstructure was not perfectly vertically oriented (see Figure S8a), and the energy profiles in Figure S8b have been slightly shifted horizontally so that the distance of 0 µm always corresponds to the centre of the supramolecular NA.

The dataset D (violet) corresponds to average values extracted from the violet hatched area in figure S8a. This area averages over 20 individual line scans.

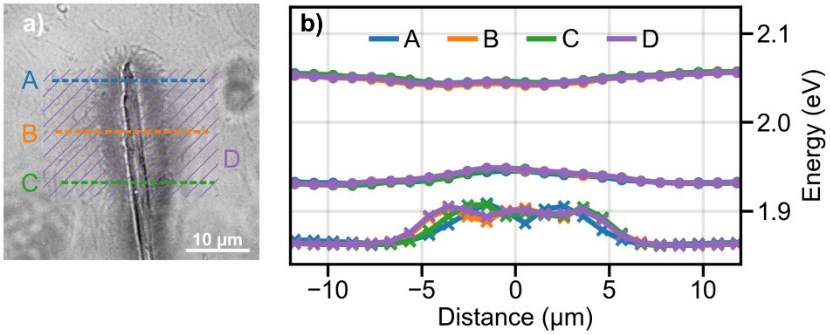

Figure S8: (a) Optical brightfield image of the NA/P3HT superstructure discussed in the main text. **(b)** Excited-state energy landscapes, analogous to figure 3b of the main text. Circles indicate the upper and lower band edge, respectively, of the *m*=0 vibronic exciton band. Crosses indicate the relaxed, lowest-energy exciton state from where emission occurs. Energy profiles were extracted along the colored lines A (blue), B (orange), and C (green) as marked in figure (a). Profile B corresponds to the data shown in figure 3b of the main text. The energy profile D (violet) corresponds to the average profile extracted from the hatched area marked with D (violet) in figure (a), corresponding to an average over 20 individual line-scans.

S6: Additional dataset

In **Figure S9**, we show the data recorded on *a different* NA/P3HT superstructure, which was prepared with a crystallisation time of 7 days. P3HT nanofibers are shorter in this sample, and the effects on free exciton bandwidth *W*, 0-0 transition energy E^{Abs}_{00}, and E^{PL}_{00} occur closer to the supramolecular NA. Yet, the gradient is again reproducibly imprinted into all nanofibres.

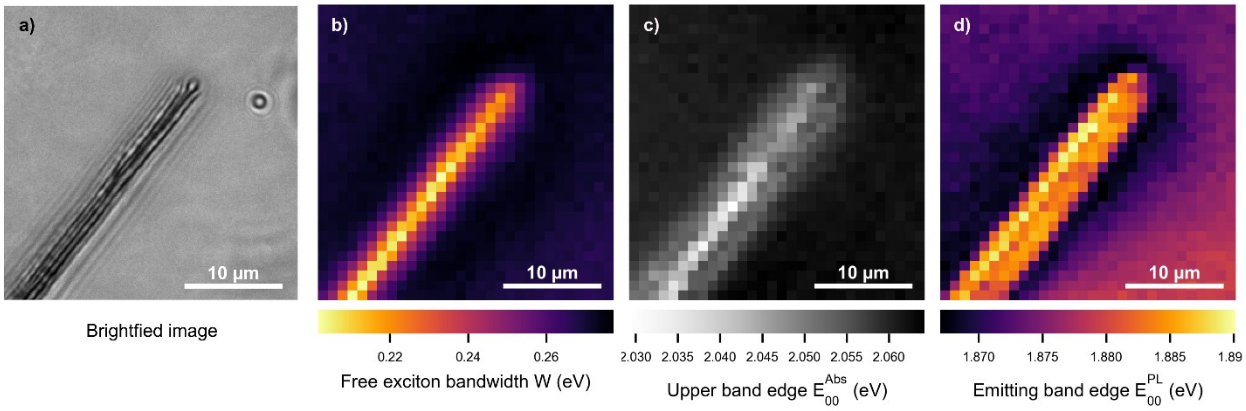

Figure S9: *Analysis of 40x40µm hyperspectral images of another NA/P3HT superstructure. a) Brightfield microscopy image. b) Map of the free exciton bandwidth W, as extracted from absorption fits. c) Map of the spectral position of the 0-0 transition E^{Abs}_{00}, as extracted from absorption fits. d) Map of the 0-0 position E^{PL}_{00} as extracted from PL fits.*

S7: References

(1) Wenzel, F. A.; Welz, H.; van der Zwan, K. P.; Stäter, S.; Kreger, K.; Hildner, R.; Senker, J.; Schmidt, H.-W. Highly Efficient Supramolecular Nucleating Agents for Poly(3-Hexylthiophene). *Macromolecules* **2022**, *55* (7), 2861–2871. https://doi.org/10.1021/acs.macromol.1c02283.

(2) Panzer, F.; Sommer, M.; Bässler, H.; Thelakkat, M.; Köhler, A. Spectroscopic Signature of Two Distinct H-Aggregate Species in Poly(3-Hexylthiophene). *Macromolecules* **2015**, *48* (5), 1543–1553. https://doi.org/10.1021/acs.macromol.5b00129.

(3) Brinkmann, M.; Wittmann, J.-C. Orientation of Regioregular Poly(3-Hexylthiophene) by Directional Solidification: A Simple Method to Reveal the Semicrystalline Structure of a Conjugated Polymer. *Advanced Materials* **2006**, *18* (7), 860–863. https://doi.org/10.1002/adma.200501838.

(4) Brinkmann, M. Structure and Morphology Control in Thin Films of Regioregular Poly(3-Hexylthiophene). *Journal of Polymer Science Part B: Polymer Physics* **2011**, *49* (17), 1218–1233. https://doi.org/10.1002/polb.22310.

(5) Liu, J.; Arif, M.; Zou, J.; Khondaker, S. I.; Zhai, L. Controlling Poly(3-Hexylthiophene) Crystal Dimension: Nanowhiskers and Nanoribbons. *Macromolecules* **2009**, *42* (24), 9390–9393. https://doi.org/10.1021/ma901955c.

(6) Gierschner, J.; Huang, Y.-S.; Van Averbeke, B.; Cornil, J.; Friend, R. H.; Beljonne, D. Excitonic versus Electronic Couplings in Molecular Assemblies: The Importance of Non-Nearest Neighbor Interactions. *The Journal of Chemical Physics* **2009**, *130* (4), 044105. https://doi.org/10.1063/1.3065267.

(7) Clark, J.; Chang, J.-F.; Spano, F. C.; Friend, R. H.; Silva, C. Determining Exciton Bandwidth and Film Microstructure in Polythiophene Films Using Linear Absorption Spectroscopy. *Applied Physics Letters* **2009**, *94* (16), 163306. https://doi.org/10.1063/1.3110904.

(8) Spano, F. C. The Spectral Signatures of Frenkel Polarons in H- and J-Aggregates. *Accounts of Chemical Research* **2010**, *43* (3), 429–439. https://doi.org/10.1021/ar900233v.

(9) Raithel, D.; Baderschneider, S.; de Queiroz, T. B.; Lohwasser, R.; Köhler, J.; Thelakkat, M.; Kümmel, S.; Hildner, R. Emitting Species of Poly(3-Hexylthiophene): From Single, Isolated Chains to Bulk. *Macromolecules* **2016**, *49* (24), 9553–9560. https://doi.org/10.1021/acs.macromol.6b02077.

(10) Raithel, D.; Simine, L.; Pickel, S.; Schötz, K.; Panzer, F.; Baderschneider, S.; Schiefer, D.; Lohwasser, R.; Köhler, J.; Thelakkat, M.; Sommer, M.; Köhler, A.; Rossky, P. J.; Hildner, R. Direct Observation of Backbone Planarization via Side-Chain Alignment in Single Bulky-Substituted Polythiophenes. *Proceedings of the National Academy of Sciences* **2018**, *115* (11), 2699–2704. https://doi.org/10.1073/pnas.1719303115.

(11) Parkinson, P.; Müller, C.; Stingelin, N.; Johnston, M. B.; Herz, L. M. Role of Ultrafast Torsional Relaxation in the Emission from Polythiophene Aggregates. *J. Phys. Chem. Lett.* **2010**, *1* (19), 2788–2792. https://doi.org/10.1021/jz101026g.
